# Comparison of the clinical outcome of endoscopic push-through myringoplasty and microscopic overlay myringoplasty: matching co-variated designs

**DOI:** 10.1186/s12893-022-01504-3

**Published:** 2022-02-11

**Authors:** Kanokkarn Mahawerawat, Pornthep Kasemsiri

**Affiliations:** 1 Department of Otorhinolaryngology, Khon Kaen Hospital, Khon Kaen, Thailand; 2 Skull Base Surgery Unit, Department of Otorhinolaryngology, Srinagarind Hospital, Faculty of Medicine, Khon Kaen University, Khon Kaen, Thailand 40002; 3 Khon Kaen Ear Hearing and Balance Research Group, Khon Kaen University, Khon Kaen, Thailand; 4 Srinagarind Minimally Invasive Surgery Center of Excellence, Faculty of Medicine, Khon Kaen University, Khon Kaen, Thailand; 5 Khon Kaen Head and Neck Oncology Research, Faculty of Medicine, Khon Kaen University, Khon Kaen, Thailand

**Keywords:** Myringoplasty, Endoscopy, Microscopy, Tympanic membrane

## Abstract

**Background:**

The conventional microscopic overlayer myringoplasty is preferred because it allows a both hands technique, not reducing middle ear space, increasing the blood supply in the repaired area, and providing graft support; however, this technique may be troublesome for the novice surgeon during tympanomeatal flap elevation. Recently, the endoscopic push-through myringoplasty technique has developed. It provides better visualization of the hidden areas and does not require raising tympanomeatal flap. Therefore, the comparison of clinical outcomes between endoscopic push-through myringoplasty and conventional microscopic overlay myringoplasty technique was investigated.

**Methods:**

A retrospective case–control hospital-based study was conducted using archival data from the patients who underwent myringoplasty between January 2015 and May 2021 at Srinagarind Hospital and Khon Kaen Hospital, Thailand. The medical records of patients who underwent endoscopic push-through technique or microscopic overlayer technique were chosen by simple randomization and matched 1:1 based on the air conduction threshold, air-bone gap, size of perforation, and experience of the surgeon. The two techniques were compared for clinical outcome success, including tympanic membrane closure, improved air conduction threshold, air-bone gap closure, and operation time duration.

**Results:**

Medical records of 70 patients were retrieved and classified into 35 patients who underwent endoscopic push-through and 35 patients who underwent microscopic overlayer myringoplasty. The size of tympanic membrane perforation and preoperative audiometry were not significantly different between both groups (p > 0.05). The postoperative outcome in endoscopic technique revealed that the air-bone gap and the success rate of tympanic membrane closure were comparable with microscopic techniques (p = 0.420 and p = 0.156, respectively). The operation time was significantly shorter in the endoscopic technique (p < 0.05). Complications were found in one patient with otitis externa in the endoscopic technique group and one patient with graft lateralization in the microscopic technique group.

**Conclusions:**

Endoscopic push-through myringoplasty is an alternative minimally invasive technique that may allow the potential outcomes comparable with the microscopic overlayer myringoplasty and with a significantly shorter operation time.

## Introduction

Chronic tympanic membrane perforation is one of the causes of middle ear infection due to loss of barrier between the external ear and middle ear; therefore, the organisms can pass through and develop middle ear infection. Thus, closure of the tympanic membrane is required to prevent infection. Myringoplasty or tympanoplasty has been introduced for the repair of tympanic membrane perforation. Since 1950, microscopy has been used to facilitate myringoplasty as the gold standard tool-assisted surgery [1] with success rates of 80–90% [2]. There are several microscopic techniques for tympanic membrane perforation closure, including the postauricular approach, transcanal approach, and end aural approach. The postauricular approach facilitates access for more difficult tympanic membrane repairs with anterior or large perforations as well as anterior bony canal overhang [3]. However, this approach may produce surgical scarring, temporary loss of cutaneous sensation [4], and malposition of the ear. In 1990, minimally invasive endoscopy was introduced for middle ear surgery [5] and allowed the surgeon to perform a transcanal approach avoiding surgical scarring and providing better visualization of hidden areas. However, there are several disadvantages including loss of depth perception, difficulty to control bleeding, and one-hand technique.

Graft placing technique is a very important factor that affects the success rate of surgical outcomes. There are several graft placing methods; however, the overlayer and underlayer are the most common method where the graft is placed above or below the fibrous annulus, respectively. The overlayer technique is preferred for closing tympanic membrane perforation as it does not reduce middle ear space, increases blood supply in the repaired area, and provides graft support [6]; however, this technique may be troublesome for the novice surgeon due to need to raise the tympanomeatel flap that may lead to bleeding during dissection. Therefore, graft placing with push-through technique via endoscopic approach was developed to avoid the tympanomeatal flap elevation step. There is a lack of comparison data between conventional microscopic overlayer myringoplasty and endoscopic push through myringoplasty; thus, this study was designed to compare the surgical outcomes between the two techniques with matching covariate design for reducing confounding factors.

## Materials and methods

### Study design

This retrospective case–control study was conducted between January 2015 and May 2021 at Srinagarind and Khon Kaen Hospitals, the two government tertiary hospitals in Khon Kaen, provincial capital of Khon Kaen Province, Northeastern Thailand. Medical records of patients who underwent myringoplasty type I with endoscopic push-through technique or microscopic overlayer technique were chosen via matched 1:1 based on the air conduction threshold, air-bone gap, tympanic membrane perforation size, and experience of the surgeon. If one endoscopic case was compatible with more than one microscopic case, simple randomization was used to select the microscopic case. Inclusion criteria included patients with a history of dry ear average of 3 months, different preoperative air conduction thresholds between two approaches ≤ 10 dB (an average pure-tone air-conduction threshold at 500–2000 Hz), and the different preoperative air-bone gap between two approaches ≤ 10 dB. Tympanic membrane perforation sizes were matched between the two groups. The perforation size was estimated with comparable a quadrant of the tympanic membrane that was divided into four quadrants; therefore, the perforation of one quadrant was estimated at approximately 25%. Subsequently, the perforation sizes were categorized as small (involving < 40% tympanic membrane), medium (involving 41–69% tympanic membrane), and large (involving ≥ 70% of the tympanic membrane). Regarding surgeons’ experience, the endoscopic push through myringoplasty was performed by young staffs (either P.K. or K.M.), and the microscopic approach being performed by last year’s residents and staff.

### Surgical technique

#### Endoscopic push-through myringoplasty

**Fig. 1:**
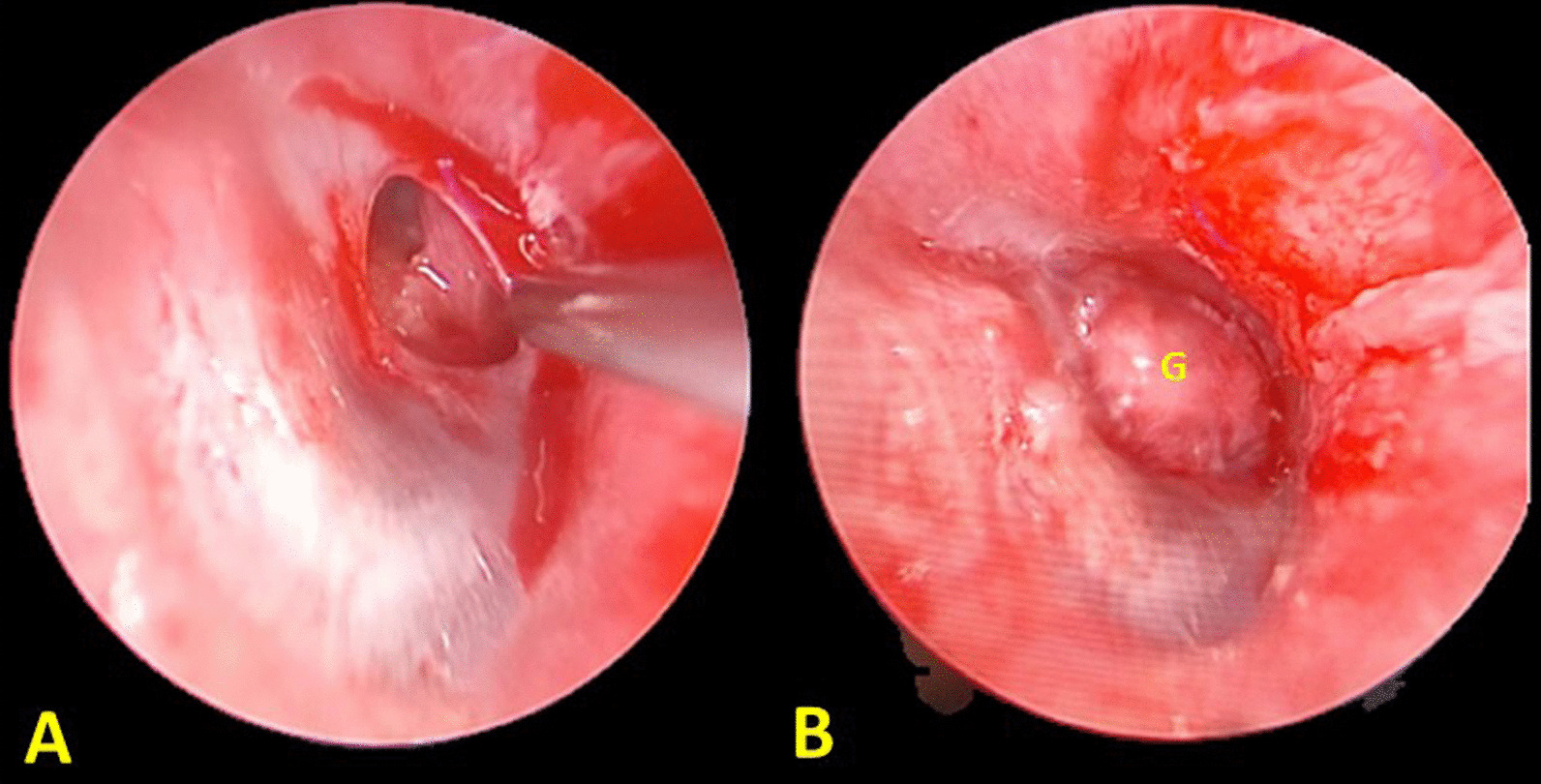
Endoscopic push-through myringoplasty was performed on the right ear. The edge of tympanic membrane perforation was trimmed (**A**). A temporalis fascia graft was harvested and inserted through the perforation as the underlayer technique **(B**). (G = temporalis fascia graft)

After standard protocol general anesthesia was induced, endoscopic myringoplasty was performed via the transcanal approach. The surgeon used a 0-degree, 3 mm diameter, 14 cm length rigid endoscope to visualize and examined the middle ear. The edge of tympanic membrane perforation was trimmed with the angle sharp pick (Fig. 1A). A temporalis fascia or tragal perichondrial graft was harvested slightly larger than perforation. Small pieces of gel foam were inserted through the perforation and tightly packed in the middle ear. Next, the graft was pushed through the perforation and placed with using the underlayer technique (Fig. 1B). Finally, the external canal was packed with gel foam.

#### Microscopic overlay myringoplasty

**Fig. 2:**
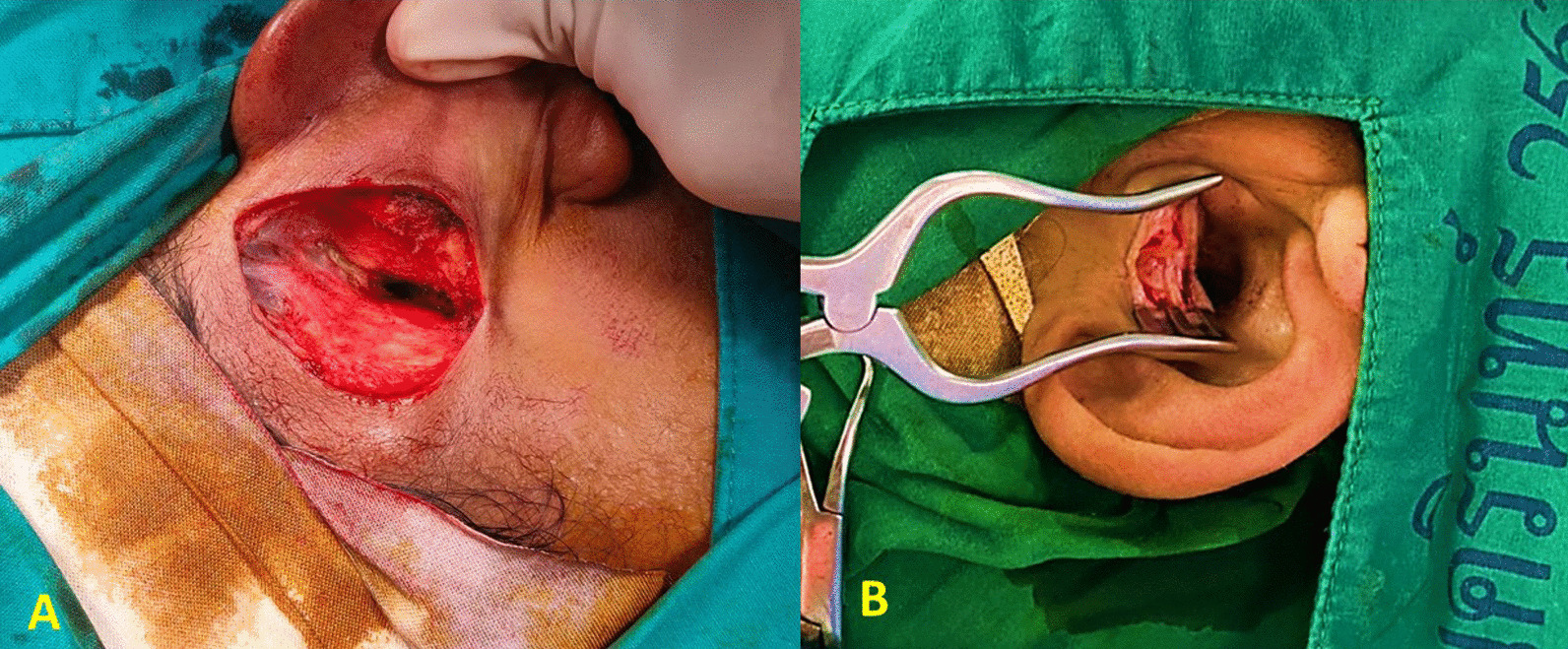
Microscopic overlayer myringoplasty may be performed via postauricular approach (**A**) or end aural approach (**B**)

After general anesthesia was induced, a local injection of adrenaline 1:100,000 combined with 2% lidocaine was given at the postauricular crease and external ear canal. A posterior auricular incision was made (Fig. 2A) and the flap was elevated and then a temporalis fascial graft was harvested. The posterior external ear canal skin was cut (1 cm average) above the remnant annulus to access the middle ear via the posterior canal wall. The end aural approach was the other technique used for microscopic myringoplasty. The skin incision was made at the external ear canal along the tympanic annulus then an incision was made (Fig. 2B) along the petrotympanic fissure for the entire length of the anteroposterior portion of the external bony ear canal. This incision was extended upward between the tragus and helix. The skin and subcutaneous were elevated and separated, then the temporalis fascial graft was harvested. Sequentially, the graft preparation was as follows. The tympanomeatal flap and the fibrous layer of the remnant tympanic membrane were carefully elevated. Gel foam was packed in the middle ear and then the graft was placed using the overlayer technique. The tympanomeatal flap and the fibrous layer of the tympanic membrane were repositioned to cover the graft. Finally, gel foam was gently packed in the external ear canal, and skin closure was then performed.

**Fig. 3:**
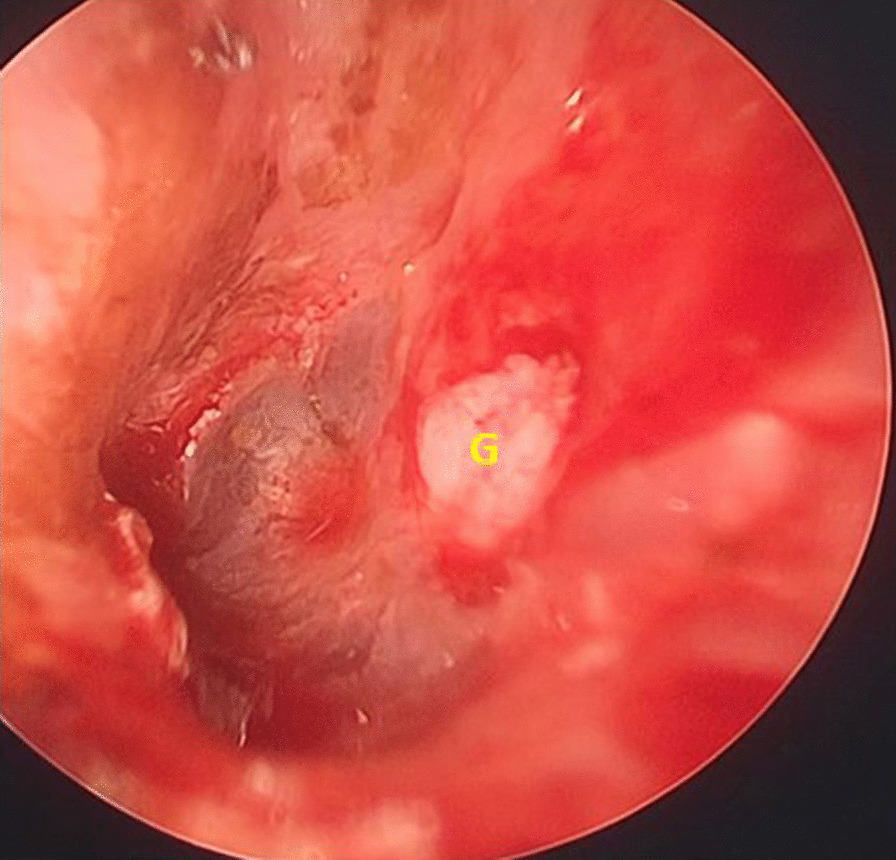
The tympanic membrane was closed by graft at the end of the 1st month postoperatively

All of the patients who underwent myringoplasty received oral and started antibiotics ear drop after surgery 24–48 h for 2 weeks. The residual gel foam in the external ear canal was removed 2 weeks post-surgery. One-month post-surgery, microscopic or endoscopic examination was conducted to assess graft uptake (Fig. 3), and hearing was assessed by audiometry.

### Statistical analysis

Sample size calculation was based on an estimated mean for the postoperative air-bone gap of microscopic overlayer myringoplasty group of 19.6 ± 10.5 dB [7], an estimated mean for postoperative air-bone gap of endoscopic push through myringoplasty group of 12.15 ± 3.98 dB [8] and 95% confidence and 2% error levels. With an acceptable equivalence margin of 5%, the calculated sample size for each group was 35 medical record charts per group.

Descriptive data were presented via percentages and mean ± SD. The independent T-test was used to analyze the correlation of clinical outcomes between endoscopic and microscopic groups that presented with continuous data in a normal distribution. However, abnormal distribution was observed in the post-operative air-bone gap outcomes between both groups; thus, this parameter was analyzed with the Mann–Whitney test. Otherwise, the outcomes between pre-and post-operation were analyzed with the Wilcoxon signed-rank test separately in each group. The Chi-square test was used to compare categorical data between both groups. A value of p < 0.05 was considered statistically significant. All data were analyzed using STATA (v 10.1: Stata Corp. 2015, Texas, USA).

### Ethical review

The study was reviewed and approved by the Human Ethics Committee of Khon Kaen University (HE641341) and the Ethics Committee of Khon Kaen Hospital (KEMOU64018).

## Results

A total of 70 patients were enrolled and assigned to the two groups, endoscopic push-through myringoplasty technique or microscopic overlayer myringoplasty technique. There were 35 ears per group for analysis. Both groups demographic data were similar (p > 0.05). There was no statistically significant difference in preoperative audiometric parameters or tympanic membrane perforation size between the groups (p > 0.05) (Table 1).

**Table 1:** Demographic data

Characteristics	Endoscopic push-through myringoplasty (N = 35)	Microscopic overlayer myringoplasty (N = 35)	p-value
Gender			
Male: n (%)	9 (25.7%)	11 (31.4%)	0.598
Female: n (%)	26 (74.3%)	24 (68.6%)	0.598
Age (mean ± SD; years)	49.4 ± 11.4	50.1 ± 11.0	0.795
Underlying disease: n (%)			
No	20 (57.1%)	25 (71.4%)	0.212
Diabetes mellitus	2 (5.7%)	5 (14.3%)	0.230
Hypertension	7 (20.0%)	5 (14.3%)	0.527
Other	13 (37.1%)	6 (17.1%)	0.059
Size of tympanic membrane perforation: n (%)			
Small (≤ 40%)	14 (40.0%)	14 (40.0%)	1.000
Medium (41–69%)	12 (34.3%)	12 (34.3%)	1.000
Large (≥ 70%)	9 (25.7%)	9(25.7%)	1.000
Anterior perforation of tympanic membrane: n (%)	22 (62.9%)	11(31.4%)	0.007
Preoperative audiometry (mean ± SD)			
Air conduction threshold (dB)	39.9 ± 13.7	40.7 ± 12.5	0.216
Bone conduction threshold (dB)	18.0 ± 7.5	19.0 ± 7.5	0.315
Air-bone gap (dB)	22.0 ± 9.9	22.0 ± 9.0	0.963
Revision case: n (%)	6 (17.1%)	3 (8.6%)	0.288

Pre-and postoperative audiometric testing for air conduction threshold and air-bone gap showed statistically significant post-surgery improvement in both groups (p < 0.05); whereas, the bone conduction threshold showed no significant different improvement after surgery in either group (p > 0.05) (Table 2). The overall success rate of tympanic membrane closure was similar for both groups (p > 0.05). Further analysis of size perforation sub-groups showed no difference in closure rate for different perforation sizes between the groups (p > 0.05). The operative time the endoscopic group was significantly shorter than the microscopic group (p < 0.05) (Table 2).

**Table 2:** Comparison of surgical outcomes and operative time in both groups

Surgical outcomes	Endoscopic push-through myringoplasty (N = 35)	Microscopic overlayer myringoplasty (N = 35)	p-value
Air conduction threshold (mean ± SD; dB)			
Preoperative	39.9 ± 13.7	40.7 ± 12.5	0.216
Postoperative	28.6 ± 14.2	33.6 ± 15.8	0.101
p-value	< 0.0001	0.002	
Bone conduction threshold (mean ± SD; dB)			
Preoperative	18.0 ± 7.5	19.0 ± 7.5	0.315
Postoperative	17.0 ± 8.3	18.0 ± 7.9	0.610
p-value	0.178	0.379	
Air-Bone gap (mean ± SD; dB)			
Preoperative	22.0 ± 9.9	22.0 ± 9.0	0.963
Postoperative	11.0 ± 9.6	16.0 ± 13.0	0.108
p-value	< 0.0001	0.006	
Overall tympanic membrane closure: n (%)	27 (77.1%)	24 (68.6%)	0.591
Tympanic membrane closure in patients with			
Small perforation (≤ 40%)	11 (31.4%)	13 (37.1%)	0.589
Medium perforation (41–69%)	10 (28.6%)	7 (20.0%%)	0.369
Large perforation (≥ 70%)	6 (17.1%)	4 (11.4%)	0.225
Operative time (mean ± SD; min)	57.8 ± 16.7	86.0 ± 33.0	< 0.001

Regarding postoperative complication, we found that one patient with acute otitis externa in the endoscopic group; whereas, one patient with graft lateralization was observed in the microscopic group. The patient with acute otitis externa was treated with oral antibiotics for 1 week with clinical improvement. The patient with graft lateralization was scheduled for revision myringoplasty.

## Discussion

The best technique for myringoplasty is still under debate because of rapidly growing novel technology leading to paradigm shifts in minimally invasive surgery. The conventional microscope has been considered the ideal surgical tool to facilitate ear surgery, whereas, the endoscope is considered a novel alternative surgical tool for minimally invasive ear surgery. Several literature reviews have shown endoscopic ear surgery is a safe procedure [9, 10].

A previous systematic review [11] compared surgical outcomes between microscopic and endoscopic myringoplasty that found a similar postoperative graft success rate between both groups (OR 0.99; p = 0.894). The postoperative air-bone gap was significantly better in the endoscopic group than the microscopic group (mean difference of 2.02; p = 0.029); however, substantial heterogeneity in surgical technique details and publication bias limited the conclusions of this study. Therefore, we conducted this study with a matching co-variated design to compare endoscopic push-through myringoplasty and conventional microscopic overlayer myringoplasty. Endoscopic push-through myringoplasty is a simple and minimally invasive technique; whereas, microscopic overlayer myringoplasty allowed the advantages including available use for perforations of varying locations and sizes (typically reserved for the complicated cases and anterior tympanic membrane perforation [12]), and avoiding chorda tympani injury. We conducted this study to expand current limited comparative research data on outcomes between endoscopic push-through and microscopic overlayer myringoplasty surgery.

Our study showed mean of air conduction threshold and air-bone gap outcomes were significantly improved after either surgical technique (p < 0.05). Tympanic membrane closure success rate was also similar between endoscopic push-through (77.1%) and microscopic overlayer myringoplasty (68.6%) (p > 0.05). The success rate of tympanic membrane closure was not statistically different between both techniques when perforation size in sub-groups were compared. These findings were similar to Plodpai Y’s study [13] that compared endoscopic and microscopic overlayer techniques and found similar success rates for tympanic membrane closure for the endoscopic and microscopic group were 97.1% and 93.3%, respectively (p = 0.60). We found the mean postoperative air-bone gap from endoscopic push-through technique (11.0 ± 9.6 dB) was better than the microscopic overlayer technique (16.0 ± 13.0 dB) but the difference was not statistically significant; whereas, Plodpai [13] showed a significantly better mean postoperative air-bone gap in the endoscopic overlayer group (5 ± 5.4 dB) than the microscopic overlayer group (10.3 ± 9.6 dB) (p = 0.01). At this point, the endoscopic technique seemed to allow better postoperative air-bone gap than microscopic overlayer myringoplasty. However, audiometric outcomes were unclear between endoscopic push-through and overlayer technique due to lack of comparison data. Several previous studies [14–16] compared the surgical outcomes between endoscopic push-through and microscopic underlayer technique. The success rate for tympanic membrane closure was 90.0–92.4% in the endoscopic push-through technique; whereas, the tympanic closure rate was 85.0–89.7% in the microscopic underlayer technique. This difference is similar to our study but the overall success rate of tympanic membrane closure was better than our series due to baseline demographic data differences including perforation sizes, anterior perforation of the tympanic membrane, and revision surgery. Approximately half of our patients presented medium to large perforation or anterior perforation that were the high-risk factors for the failure of the tympanic membrane closure. Although microscopic overlayer technique was considered as ideal for repairing any size of perforations due to providing high vascularization, and secure graft placement, the microscopic technique may be difficult to repair anterior perforation because the microscope magnified the surgical field on a straight line. Therefore, the anterior edge of the perforation and annulus may be obscured by the prominent anterior canal wall. Furthermore, the experience of the surgeon was one of the confounding factors. In our series, the junior staffs were just starting to use myringoplasty with endoscopic push-through technique; whereas, some patients in microscopic overlayer technique were operated by residents. Thus, their learning curves may have affected our surgical outcomes. The other possible confounding factors (middle ear status and eustachian function) would affect the tympanic membrane closure outcome, also. Unfortunately, these confounding factors were unavailable retrieving from our patients’ medical record chart that was the limitation of this study.

However, the previous studies showed no significant between groups difference in postoperative air-bone gap outcomes that were similar to our study. Furthermore, the operation time was observed that a significantly shorter in the endoscopic technique group (p < 0.05).

Regarding complications, we found few minor complications including acute otitis externa in 1 endoscopic group patient and 1 graft lateralization in the microscopic group. A serious complication concern has been raised that heat from the endoscopic light source could damage the inner ear. However, no patients in the endoscopic group complained about vertigo, dizziness, or nystagmus. And we found no postoperative bone conduction threshold reduction post-surgery; therefore, we consider the heat from the endoscopic light source didn’t damage the inner ear in our series. To obviate inner ear heat damage, during surgery we agree with Kozin et al. [17] to use submaximal light intensity and frequent repositioning of the endoscope.

A strength of our study was the matching covariate parameters for obviating confounding factors. However, a limitation was the short-term follow-up in the endoscopic group (53.1 ± 52.1 days) compared to the microscopic group (87.9 ± 45.3 days). We suggest further research should investigate longer surgical and postoperative audiometric outcomes.

## Conclusion

Endoscopic push-through myringoplasty is an alternative minimally invasive technique to microscopic myringoplasty. We found postoperative audiometry outcomes and successful tympanic membrane closure rates may be comparable to microscopic overlayer myringoplasty; however, surgeons should be concerned about some confounding factors that were still not to be investigated due to the retrospective nature of the study design. Furthermore, the endoscopic push-through technique provided significantly shorter operation times and few minor complications.

## Data Availability

The datasets used and/or analyzed during the current study are available from the corresponding author on reasonable request.
